# Systematic cascade screening in the Danish Fabry Disease Centre: 20 years of a national single-centre experience

**DOI:** 10.1371/journal.pone.0277767

**Published:** 2022-11-16

**Authors:** Grigoris Effraimidis, Åse Krogh Rasmussen, Morten Dunoe, Lis F. Hasholt, Flemming Wibrand, Soren S. Sorensen, Allan M. Lund, Lars Kober, Henning Bundgaard, Puriya D. W. Yazdanfard, Peter Oturai, Vibeke A. Larsen, Vitor Hugo Fraga de Abreu, Lotte Hahn Enevoldsen, Tatiana Kristensen, Kirsten Svenstrup, Margrethe Bastholm Bille, Farah Arif, Mette Mogensen, Mads Klokker, Vibeke Backer, Caroline Kistorp, Ulla Feldt-Rasmussen

**Affiliations:** 1 Department of Endocrinology and Metabolism, Rigshospitalet (Copenhagen University Hospital), Copenhagen, Denmark; 2 Department of Endocrinology and Metabolic Diseases, Larissa University Hospital, Faculty of Medicine, School of Health Sciences, University of Thessaly, Larissa, Greece; 3 Department of Clinical Genetics, Rigshospitalet (Copenhagen University Hospital), Copenhagen, Denmark; 4 Institute of Cellular and Molecular Medicine, University of Copenhagen, Copenhagen, Denmark; 5 Department of Nephrology, Rigshospitalet (Copenhagen University Hospital), Copenhagen, Denmark; 6 Department of Clinical Medicine, Faculty of Health and Clinical Sciences, Copenhagen University, Copenhagen, Denmark; 7 Centre of Inherited Metabolic Diseases, Departments of Clinical Genetics and Pediatrics, Rigshospitalet (Copenhagen University Hospital), Copenhagen, Denmark; 8 Department of Cardiology, The Heart Center, Rigshospitalet (Copenhagen University Hospital), Copenhagen, Denmark; 9 Department of Clinical Physiology and Nuclear Medicine, Rigshospitalet (Copenhagen University Hospital), Copenhagen, Denmark; 10 Department of Radiology, Rigshospitalet (Copenhagen University Hospital), Copenhagen, Denmark; 11 Department of Neurology, Copenhagen Neuromuscular Center, Rigshospitalet (Copenhagen University Hospital), Copenhagen, Denmark; 12 Department of Clinical Neurophysiology, Rigshospitalet (Copenhagen University Hospital), Copenhagen, Denmark; 13 Department of Ophthalmology, Rigshospitalet-Glostrup (Copenhagen University Hospital), Copenhagen, Denmark; 14 Department of Dermatology, Bispebjerg Hospital, Copenhagen University Hospitals, Copenhagen, Denmark; 15 Department of Otorhinolaryngology and Head and Neck Surgery and Audiology, Rigshospitalet (Copenhagen University Hospital), Copenhagen, Denmark; University of Ferrara: Universita degli Studi di Ferrara, ITALY

## Abstract

The lysosomal storage disorder Fabry disease is caused by deficient or absent activity of the *GLA* gene enzyme α-galactosidase A. In the present study we present the molecular and biochemical data of the Danish Fabry cohort and report 20 years’ (2001–2020) experience in cascade genetic screening at the Danish National Fabry Disease Center. The Danish Fabry cohort consisted of 26 families, 18 index patients (9 males and 9 females, no available data for 8 index-patients) and 97 family members with a pathogenic *GLA* variant identified by cascade genetic testing (30 males and 67 females). Fourteen patients (5 males and 9 females; mean age of death 47.0 and 64.8 years respectively) died during follow-up. The completeness of the Fabry patient identification in the country has resulted in a cohort of balanced genotypes according to gender (twice number of females compared to males), indicating that the cohort was not biased by referral, and further resulted in earlier diagnosis of the disease by a lower age at diagnosis in family members compared to index-patients (mean age at diagnosis: index-patients 42.2 vs. family members 26.0 years). Six previously unreported disease-causing variants in the *GLA* gene were discovered. The nationwide screening and registration of Fabry disease families provide a unique possibility to establish a complete cohort of Fabry patients and to advance current knowledge of this inherited rare lysosomal storage disorder.

## Introduction

Fabry or Anderson-Fabry disease (OMIM #301500) is a rare underdiagnosed X-linked lysosomal disorder resulting from either deficient or absent activity of the lysosomal enzyme α-galactosidase A (α-galA) [1], with universal accumulation of glycosphingolipids, mainly globotriaosylceramide (Gb_3_), in lysosomes, resulting in a multisystemic disorder [2]. α-galA is encoded by the *GLA* gene (HUGO Gene Nomenclature Committee ID: 4296; Gene Entrez: 2717; NCBI reference sequence: NM_000169.2).

Fabry disease was first independently described in 1898 by the German dermatologist Johannes Fabry and the English surgeon William Anderson [3, 4]. The “familial” clustering and inherited nature of the disease were recognized in 1947 [5]. Subsequently, accumulation of Gb_3_, and galabiosylceramide inside lysosomes as well as involvement of α-galA in Gb_3_ metabolism were identified [1, 6, 7]. Introduction of an assay determining α-galA activity and identification of the genomic sequence encoding the enzyme (*GLA*), rendered biochemical and molecular confirmation of Fabry disease feasible [8–10]. Variants of the *GLA* gene were initially reported in 1989 [11].

The disease phenotype can be classified into a severe, classical phenotype, and a generally milder nonclassical, late-onset phenotype. Hemizygous males with pathogenic *GLA* variants associated with the classic phenotype have little or absent α-galA activity, marked accumulation of Gb_3_ and globotriaosylsphingosine (lyso-Gb_3_) in tissues and organs and clinical manifestations typically appearing in childhood or adolescence. In contrast, hemizygous males with the late-onset phenotypes have residual α-galA activity, and do not show the early manifestations of classical Fabry disease. Heterozygous females with pathogenic *GLA* variants may be asymptomatic or develop clinical manifestations as severe as those in males early or at a late point in life.

The first report of Fabry patients in Denmark published in 1972 [12], included a family with five male members with pain and/or peripheral paresthesia provoked by fever or overheating, angiokeratomas, proteinuria, involvement of the central nervous system, cardiac involvement and the characteristic corneal opacities. Three of the five patients died at young age (54, 33 and 38 years), and the remaining two (17 and 33 years old), were seriously affected [13]. Subsequently, when α-galA activity measurements became available in Denmark in the early 1970s, the Danish Fabry patients could be diagnosed biochemically. Nevertheless, the diagnosis of female heterozygotes remained challenging. If the X chromosome with the abnormal allele is predominantly inactivated obligate heterozygotes with no or mild symptoms could have α-galA in the normal range. The diagnostic issue was resolved in the mid-1990s, when sequencing of the *GLA* gene was introduced allowing specific identification of the disease-causing variants in the Danish patients [14]. Nevertheless, the classification of *GLA* variants of yet unknown significance as (likely) pathogenic or (likely) benign variants is still clinically challenging and requires further clinical, biochemical and, in some cases, histological investigations.

The production of α-galA by genetic engineering for direct enzyme-substitution therapy in 2001 launched a new era in the treatment of Fabry disease. The Institute of Medical Genetics at Copenhagen University, transferred the known Fabry families to a clinical facility, Department of Endocrinology and Metabolism, Rigshospitalet, which has since served as the National Danish Fabry referral center, being specialized and licensed to treat patients with multi-organ involvement as well as inherited endocrine disorders, such as diabetes mellitus, adrenal and pituitary diseases, and multiple endocrine neoplasias [15]. Children with Fabry disease are treated in Centre for Inherited Metabolic Diseases, Departments of Clinical Genetics and Pediatrics, Rigshospitalet in collaboration with above department.

Cascade screening is a systematic process offering genetic counseling and testing of relatives of an index-patient for identification of individuals at risk for a hereditary condition based on the *a priori* likelihood of a positive test [16, 17]. Fabry disease as an X-linked disorder has a high penetrance, though not 100% in female heterozygotes [18]. The X-linked nature of Fabry disease is suitable for an efficient, high diagnostic yield cascade screening over at least three generations surrounding an index-patient. In the present study, we summarize and present the molecular and biochemical data of the Fabry patients from the Danish National Fabry Disease Center spanning 20 years of screening experience.

## Materials and methods

### Study design

All Fabry patients diagnosed and followed at the Danish Fabry Disease Centre, Department of Endocrinology and Metabolism, and Centre for Inherited Metabolic Diseases, Departments of Pediatrics and Clinical Genetics, Copenhagen University Hospital, Rigshospitalet between June 2001 and June 2020 were included in the study. We also obtained data, when possible, from studies performed in the Fabry patients before 2001 at Institute of Medical Genetics, Copenhagen University. The diagnosis of hemizygous males was confirmed by identification of the enzyme deficiency and the pathogenic *GLA* gene variant. Heterozygous females were diagnosed by demonstrating the pathogenic *GLA* gene variant. A *GLA* gene variant was classified as pathogenic based on its presence in the *GLA*-specific databases [19, 20]. In principle, only male family members served to determine or confirm the phenotype and thus the clinical impact of the family variant (classical or late onset).

The Kingdom of Denmark consists of Denmark, Greenland and the Faroe Islands (with a population of 5.8 million, 56,648 and 50,844 inhabitants, respectively) [21–23]; thus, patients living in Greenland and the Faroe Islands are entitled to be referred to Danish hospital expertise at a tertiary referral center, the Danish Fabry Disease Centre.

### Cascade screening strategy

The genetically verified families transferred to Department of Medical Endocrinology and Metabolism had all received prior genetic counseling and were offered assessment of the phenotypic clinical diagnosis of Fabry disease. In all other index-cases with suspicion of the disease, genetic counseling was offered before testing for the variant. In recent years, the genetic diagnosis was often obtained via the use of gene panels for specific organ manifestations such as inherited causes of cardiomyopathy. In such cases genetic counseling was done after referral to the National Fabry Centre. At the first visit a three-generation pedigree of the family was drawn with identification of relatives to be offered cascade screening. The index-cases/family members with pathogenic *GLA* variant were subsequently encouraged to contact their relatives and transfer the information regarding the genetic Fabry disease diagnosis. Afterwards the relatives were contacted and offered genetic counseling. Family members eligible to cascade screening were initially informed by the index patient and the Fabry center contacted the family members if consent was obtained. However, in 2012 the Danish Council on Ethics has introduced five criteria for unsolicited contact with relatives [24], which are met in the case of Fabry disease and therefore our center is obliged to disclose the information to the family members in case the patient with the pathogenic *GLA* variant is not willing to do so. Only one confirmed index-case, diagnosed before 2012, refused to inform his/her family members, as well as any further contact with him/her.

**Fig 1:**
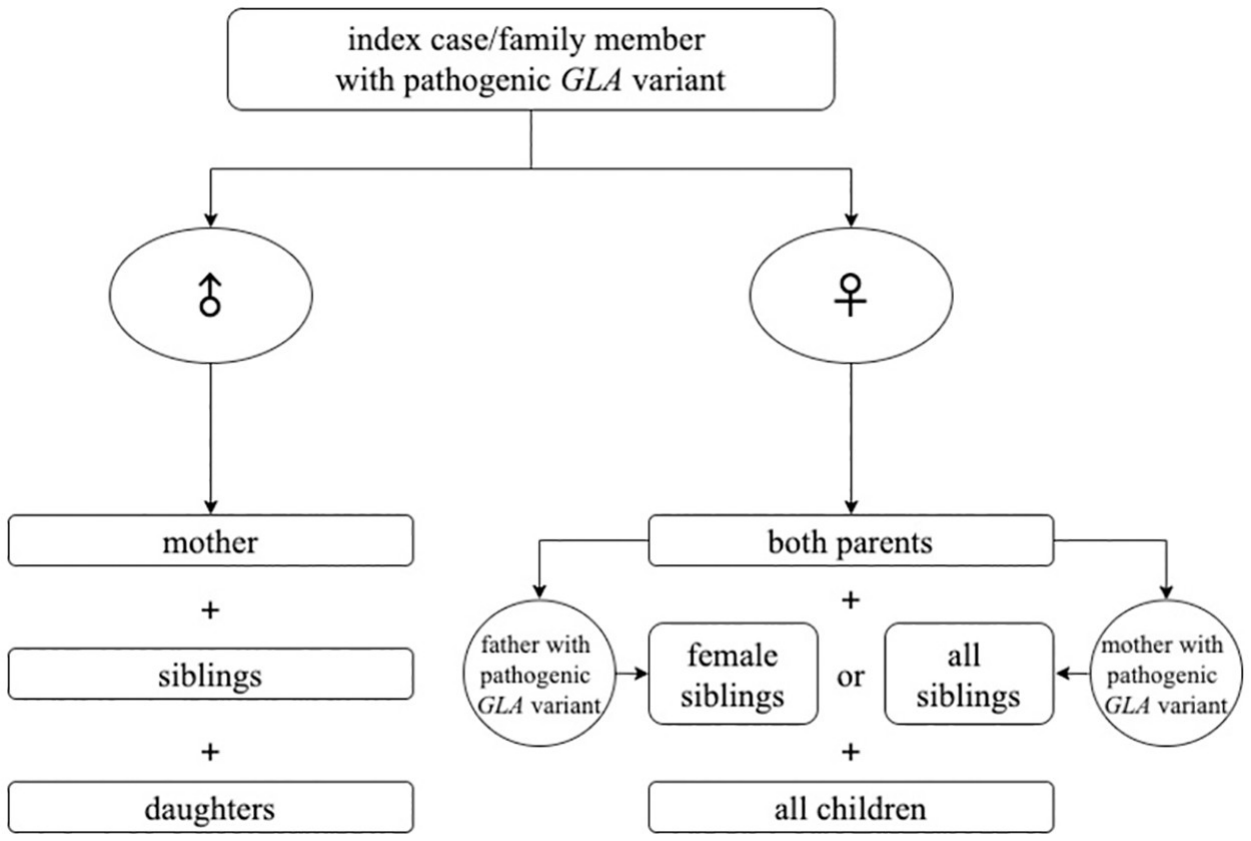
Flow chart of the principle of the procedure of the cascade screening. Cascade screening begins once an index-case has been identified. Three generations surrounding the index-case are genetically screened. Each time a family member with a pathogenic *GLA* variant is identified, the procedure is repeated. When the index-case/family member with a pathogenic *GLA* variant was male, his mother, his daughter and all his siblings were offered testing. When the index-case/family member with a pathogenic *GLA* variant was female, both her parents, and all her children were offered testing and depending on the parents’ result, either only her female siblings in case of a father with pathogenic *GLA* variant or all her siblings in case of a mother with pathogenic *GLA* variant were tested (♂ = male, ♀ = female).

The following procedure was utilized for the identification of relatives at risk of Fabry disease (Fig 1): (i) In male patients with the pathogenic variant, his mother, daughters and siblings were offered genetic testing; (ii) In females with the pathogenic variant, both her parents, and all her children were offered genetic testing and finally, depending on the parents’ results, either only her female siblings (variant inherited from father) or all her siblings (variant inherited from mother) were offered genetic testing. This process was repeated for each single family member identified with the pathogenic variant. All relatives at risk were offered a specialist physician consultation regarding the natural history, symptoms, management and treatment options of the disease before DNA screening. The genetically confirmed Fabry patients were included in the Danish Fabry Disease Database. Family members that have been tested and found not to harbor a pathogenic variant and those that refused screening have not been registered in the database due to legal restrictions.

### Evaluation of phenotype

All family members with a pathogenic *GLA* variant, independent of gender underwent comprehensive assessment of clinical symptoms and organ manifestations immediately after the genetic diagnosis was obtained. This included the following: A complete medical history and physical examination to assess features such as “Fabry facial features”, dermatologic evaluation for associated skin lesions (angiokeratomas), acroparesthesias/neuropathic pain; cardiological work-up with electrocardiography, echocardiography and later Holter monitoring; nephrology evaluation with related biochemical investigations including urinary albumin excretion, measured and estimated glomerular filtration rate; brain magnetic resonance imaging evaluation for presence of lacunar infarcts; ophthalmological evaluation for orbital traits associated with Fabry disease (cornea verticillata, retinal vessel tortuosity); sweat test for the evaluation of anhidrosis or hypohidrosis; lung function test; audiogram for the evaluation of hearing thresholds. Therefore, due to the wide spectrum of clinical manifestations, Fabry patients are followed by the Copenhagen Fabry Multidisciplinary Team, consisting of doctors and nurses from a number of different specialities (cardiologist, nephrologists, ophthalmologists, neurologists, paediatricians, radiologists, dermatologists, gastroenterologists, pulmonologists, audiology specialist, psychiatrists, genetic counselors, primary care providers, clinical nurse specialists).

### *GLA* gene analysis

Genetic testing was initially centralized in a research setting at the Department of Medical Biochemistry and Genetics, University of Copenhagen. Through 2005–8 the analysis was gradually transferred to a clinical setting at the Department for Clinical Genetics at Copenhagen University Hospital. Over time a variety of different techniques have been used for variant detection [11, 25–29]. The discovered variants were classified according to severity as classical or late-onset by the gene-specific databases when they were known [19, 20]. In case the identified *GLA* variant was absent from the gene-specific databases, the hemizygous male family members served to classify the *GLA* variant by phenotype assessment. NM_000169.3 was used as reference sequence.

### Measurement of α-galactosidase A activity in leukocytes

α-GalA activity was determined in mixed leukocytes using a fluorimetric assay with 4-methylumbelliferyl-α-D-galactopyranoside as substrate [26, 30, 31]. The fluorescence intensity was measured in a Fluostar Optima plate reader (BMG Labtech) at excitation and emission wavelengths of 360 nm and 450 nm, respectively.

### Measurement of plasma Gb_3_ and Lyso-Gb_3_ and urine Gb_3_

Gb_3_ measurements were performed at Sahlgrenska University Hospital, Sweden up to 2006 by densitometric evaluation after high performance thin layer chromatography for plasma Gb_3_ and orcinol detection in the case of urine Gb_3_ [32] and afterwards in Genzyme’s laboratories, using a rapid liquid chromatography with tandem mass spectrometry LC/MS/MS method for quantification of total plasma- lyso and urine Gb_3_ [33]. Gb_3_ units from Sahlgrenska were given in mg/L (reference: 1.6–3.3) while more recent measurements from Genzyme were in μmol/L (reference: <7.0). The calibration of methods has changed over time, but no attempts were made to correct values for unification. Therefore, each individual measurement was compared to the respective method- and laboratory derived normal reference range. Lyso-Gb_3_ was not measured in all patients before treatment start, because these measurements were not available routinely in 2001. Early participants had the measurements later in the course of the disease either during treatment or untreated. Lyso-Gb_3_ was only measured in Genzyme’s laboratory.

### Statistical analysis

Due to the nature of the data as well as low numbers in all subgroups, descriptive statistics were computed as mean (range and/or standard deviation) and/or median (range and/or interquartile range) for continuous variables and as counts and percentages for categorical variables.

### Ethics

The study was approved by the Danish Health and Medicine Authority (3-3013-667/1/), the Regional Health Research Ethics Committee (H-3-2014-FSP8) and the Danish Data Protection Agency (2014-641-0055).

## Results

### Patients—Cascade screening

A total of 115 patients were included in the Danish Fabry Disease Centre up to June 2020, born between 1936 and 2019, of whom 39 were males and 76 females. The 115 patients belong to 26 families. During the follow up period all patients resided in Denmark, Greenland or the Faroe Islands. Fourteen patients (5 males and 9 females) died during follow-up. Mean age of death for all causes was 58.5 (range 36.6–77.2) years in all patients, 47.0 (range 36.6–51.9) years in males and 64.8 (range 47.5–77.2) years in females. One hundred and one patients (34 males and 67 females) were alive at the end of the follow up period. The prevalence rate of Fabry disease in the Danish population is estimated to be 1:58000 (males 1:85000, females 1:44000). In general, family members complied to the genetic cascade screening, and only six abstained from further follow-up.

### Pathogenic *GLA* variants

**Fig 2:**
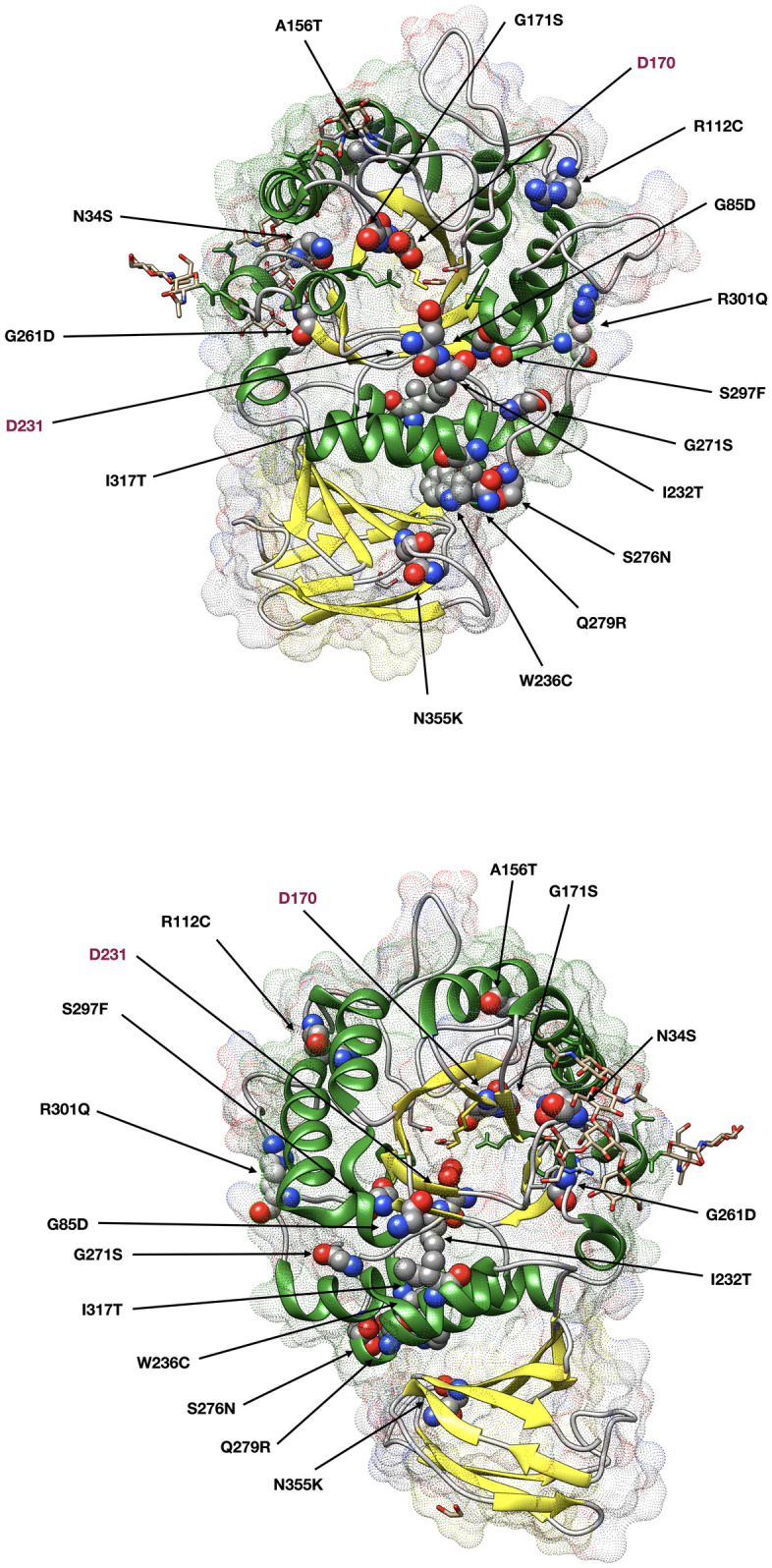
Structure of human *GLA* and positions of the amino acid substitutions resulting from the missense pathogenic variants identified in the Danish Fabry patients. The backbone is displayed as a ribbon model, and the ligand and sugars as a stick model. The amino acids involved in the substitutions and the catalytic residues (D170 and D231) are indicated as a space-filling calotte (Corey-Pauling-Koltun CPK) model. Front view (top) and back view (bottom).

In total 24 different pathogenic *GLA* variants were identified (Table 1); 15 missense variants (Fig 2), 4 variants were predicted to introduce a premature stop codon, one indel variant (c.369+3_c.547+954del4096insT), one splice site deletion (c.999+1del), one deletion (c.885del) and one duplication (c.864dup) both leading to a frameshift. Two families shared the p.N34S and two the p.R301X variants, without any recognized relationship between the families. Five of the Danish pathogenic gene variants have not been registered in the fabry-database.org [19] nor in the International Fabry Disease Genotype-Phenotype Database [20] and another one does not appear in the International Fabry Disease Genotype-Phenotype Database (Table 1).

**Table 1:** The 26 families, their 115 Fabry disease patients and their corresponding *GLA*-gene variants of the Danish Fabry disease register. Type of the *GLA* gene pathogenic variants, protein nomenclature, colloquial nomenclature, coding sequence (according to http://varnomen.hgvs.org), site of mutation and genotype classification (according to International Fabry Disease Genotype-Phenotype Database (dbFGP) http://dbfgp.org/dbFgp/fabry/ and the http://fabry-database.org) are presented. Sex, age (in years) and the primary clinical manifestation at Fabry disease diagnosis of the index-cases are presented.

Family	Amino acid change		Nucleotide change	Exon	Likely phaenotype	N (Deceased)			Index-cases	
ID						all	male	female	sex (age)	primary manifestation at diagnosis
	***Missense***									
1	p.Asn34Ser	N34S	c.101A>G	1	Classic	4	2	2	F (27)	neuropathic pain
2	p.Asn34Ser	N34S	c.101A>G	1	Classic	4(1)	2 (1)	2	M (?)	N/A deceased
3	p.Gly85Asp	G85D	c.254G>A	2	Classic	26(7)	8 (2)	18 (5)	F (?)	N/A deceased
4	p.Arg112Cys	R112C	c.334C>T	2	Classic	15	3	12	M (12)	neuropathic pain
5	p.Ala156Thr	A156T	c.466G>A	3	Classic	13(3)	3 (1)	10 (2)	M (33)	N/A deceased
6	p.Gly171Ser	G171S	c.511G>A	3	Late-onset	2	1	1	M (28)	nephropathy
7	p.Ile232Thr	I232T	c.695T>C	5	Likely classic	10(1)	4	6 (1)	M (36)	proteinuria
8	p.Trp236Cys	W236C	c.708G>C	5	Classic	3	2	1	F (51)	cardiomyopathy
9	p.Gly261Asp	G261D	c.782G>A	5	Classic	6	2	4	F (68)	cardiomyopathy
10	p.Gly271Ser	G271S	c.811G>A	6	Classic	4(1)	1	3 (1)	F (66)	cardiomyopathy
11	p.Ser276Asn	S276N	c.827G>A	6	Classic	1	1	-	F (?)^†^	cardiomyopathy
12	p.Gln279Arg	Q279R	c.836A>G	6	Classic	1	1	-	M (54)	cardiomyopathy
13	p.Ser297Phe	S297F	c.890C>T	6	Classic	2	1	1	F (73)	cardiomyopathy
14	p.Arg301Gln	R301Q	c.902G>A	6	Late-onset ^¶¶^	2	-	2	N/A	
15	p.Ile317Thr	I317T	c.950T>C	6	Classic	2	1	1	M (40)	cardiomyopathy, nephropathy, neuropathic pain
16	p.Asn355Lys	N355K	c.1065C>A	7	Classic	2	1	1	M (36)	neuropathic pain
	***Nonsense***									
17	p.Lys168Ter	K168*	c.502A>T	3	Novel^¶^	1	-	1	F (63)^†††^	cardiomyopathy
18	p.Arg227Ter	R227*	c.679C>T	5	Classic	2	-	2	M (?)	N/A deceased
19	p.Arg301Ter	R301*	c.901C>T	6	Classic	3	-	3	F (53)	cardiomyopathy
20	p.Arg301Ter	R301*	c.901C>T	6	Classic	1	-	1	M ^††^	non-Danish
21	p.Arg342Ter	R342*	c.1024C>T	7	Classic	4	1	3	M (33)	neuropathic pain
	***Other***									
22	(large deletion)		c.195-?_801 +? del	2–5	Novel^¶^	1	1	-	M (47)	cardiomyopathy
23	(deletion-insertion)		c.369+3_c.547+954del4096insT	3–4	Classic	1	-	1	F (16)	migraine
24	p.Phe295Leufs*22 (deletion-frameshift)		c.885del	6	Novel^¶^	3(1)	2 (1)	1	M (10)	neuropathic pain
25	(splice site deletion exon 6 skipping)		c.999+1del*		Novel^¶^	1	1	-	M ^††^	non-Danish
26	p.Ile289Tyrfs*10 (duplication-frameshift)		c.864dup	6	Novel^¶^	1	1	-	M ^††^	non-Danish
	Total					115	39	76		

^¶^ Novel variants that have not previously been reported in the literature nor in the Fabry Genotype-Phenotype Databases (International Fabry Disease Genotype-Phenotype Database (dbFGP) (http://dbfgp.org/dbFgp/fabry/) and http://fabry-database.org)

^¶¶^ Reference 25

^†^ never referred to the Department

^††^ does not live in Denmark

^†††^ no contact with the family

^?^ unknown

N/A not available

### Index-patients

Three patients were not of Danish origin: one male was from Middle East, one male from the United States of America, and one female from South America. None of the index-patients in these three families were diagnosed in Denmark and therefore not registered in the Danish Fabry Disease Centre (Table 1). The remaining 23 index-patients were of native Danish origin. We could not with certainty obtain data regarding the index-patients of four families as they died before 2001. One index-patient did not want baseline assessment, further contact and follow-up and refused to disclose the information to his/her family members. Another index-patient did not want further contact and follow-up after being evaluated at baseline but he/she informed his/her family members. The mean follow-up period of the living index-cases was 8.4 years (range 0.3–19.5 years).

From the 18 known index-cases, 9 were males and 9 were females, with a mean age (±standard deviation) at referral 32.9±14.6 and 51.5±19.0 years, respectively (range: 10.0–54.0 and 15.9–72.7 years, respectively). One female index-patient died during the follow-up period at the age of 71.6 years. The mean age (±standard deviation) at the end of the follow-up period for the alive male and female index-patients was 42.0±13.3 and 57.2±13.6 years, respectively (range: 19.3–61.0 and 30.4–73.8 years, respectively) (Table 2). The mean age of the family members was substantially lower than that of the index-patients especially at diagnosis but also at follow-up (Table 2).

**Table 2:** Illustration of the affected individuals of the Danish Fabry cohort at diagnosis and/or baseline assessment from 2001 onwards and at the end of the study period. Index patients and their family members were described in terms of age at diagnosis, sex, dead/alive at end of study, age at end of the study or age at death, no contact and first organ manifestation of the index patients.

	Index-cases	Family members
	(n = 18)	(n = 97)
Available data N	18	61
Age at diagnosis (years)		
Mean (SD)	42.2 (19.0)	26.0 (19.4)
Range	10.0–72.1	0.0–72.5
Sex (M/F)	9/9	30/67
Deceased		
N	1	13
Sex (M/F)	-/1	5/8
Age at diagnosis (years) Mean (SD)	66.4	
Age at diagnosis (years) Range		
Age of death Mean (years) (SD)	71.6	57.4 (12.0)
Age of death (years) Range	-	36.5–77.2
Alive		
N	17	84
Sex (M/F)	8/9	59/25
Age at diagnosis (years) Mean (SD)	40.8 (18.6)	26.0 (19.4)
Age at diagnosis (years) Range	10.0–72.7	0.0–72.5
Age at end follow-up (years) Mean (SD)	49.2 (15.2)	42.0 (18.8)
Age at end follow-up (years) Range	19.3–73.8	0.7–83.9
First manifestation		
Cardiomyopathy	8	
Neuropathic pain	5	
Kidney disease/proteinuria	2	
Migraine	1	
Cardiomyopathy, neuropathic pain, kidney	1	
Unknown	1	

Index-patients:17 + 3 unknown from abroad + 1 with no contact + 4 died before 2001 + 1 died after 2001 = 26 families

Cohort: 84 relatives alive + 18 index-patients (17 alive + 1 dead) + 13 dead (1 dead index-patient) contact = 115 patients

M: male; F: female; SD: Standard deviation

Index-cases were referred to the Department from cardiologists, nephrologists and neurologists. Eight were investigated for Fabry disease due to cardiomyopathy, five due to neuropathic pain, two due to kidney disease, one for migraine and one had cardiomyopathy, kidney disease and neuropathic pain. The reason for referral was unknown for one index-patient.

### α-galactosidase A activity

**Fig 3:**
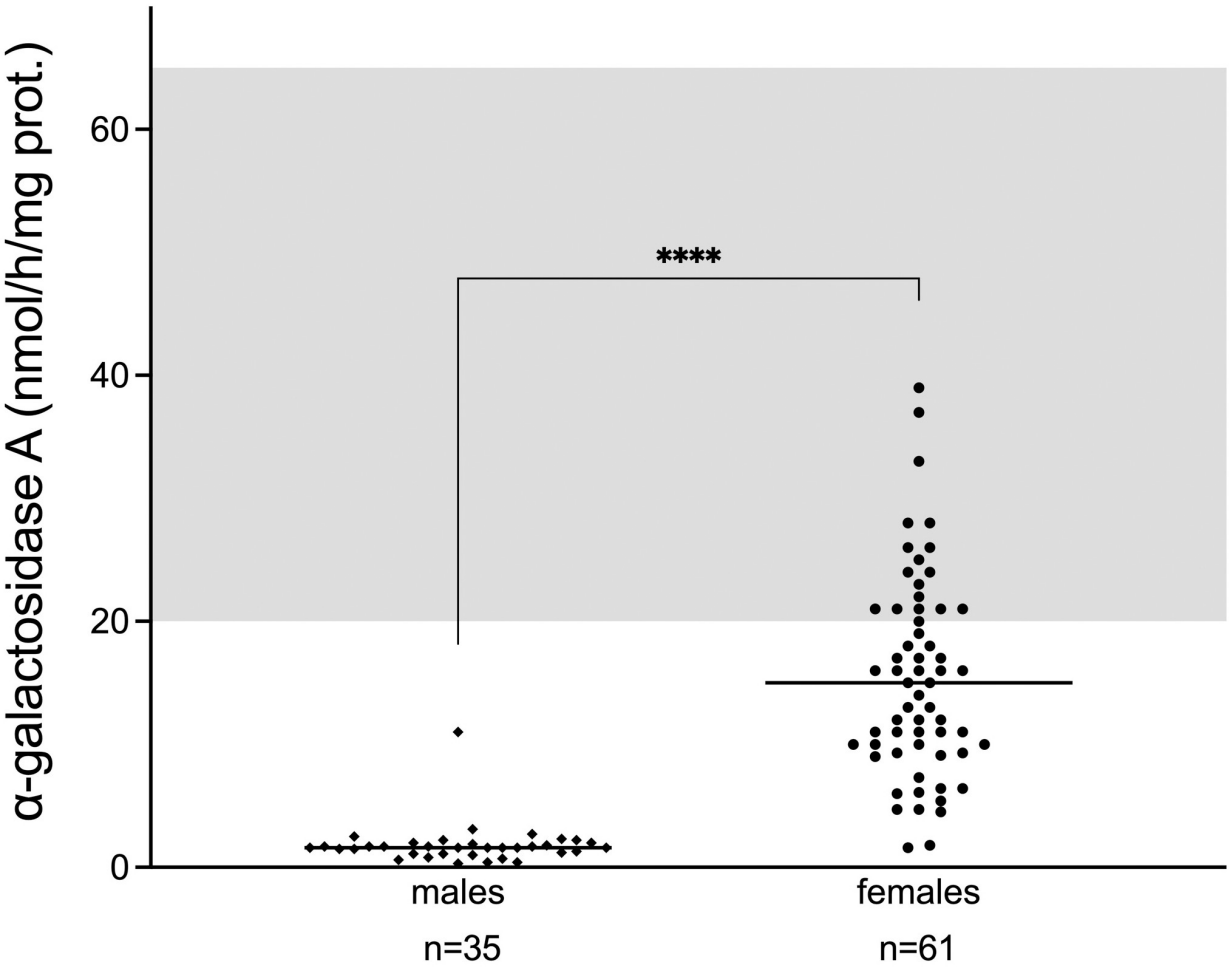
Residual α-galactosidase A activity in Fabry males and females. Activity is expressed, as nmol/h/mg protein. Grey area represents the normal α-galactosidase A activity range (20–65 nmol/h/mg protein). Available measurements in n = 35/39 males and females n = 61/76 females. ****: p-value <0.001.

Enzyme activity in leukocytes was available in 35 (90%) male and 61(80%) female patients. All males had low to absent leukocyte α-galA activity (Fig 3). The median enzyme activity in males was 1.6 nmol/h/mg protein (25th–75th percentile: 1.1–2.0 nmol/h/mg protein; range: 0.3–11.0 nmol/h/mg protein; normal α-galactosidase A activity range: 20–65 nmol/h/mg protein), whereas in female patients, the enzyme activity showed a wide range (1.6–39.0 nmol/h/mg protein; normal α-galactosidase A activity range: 20–65 nmol/h/mg protein). Of the 61 female patients tested, 17 (28%) presented with normal enzyme activity at diagnosis. The median enzyme activity in females was 15 nmol/h/mg protein (25th–75th percentile: 10–21 nmol/h/mg protein). No statistically significant difference in enzyme activity was found between females with missense and nonsense mutations (n = 52 vs. 7; median: 15.0 vs. 17.0 nmol/h/mg protein, respectively), but numbers were too small for reliable conclusions to be drawn. Comparison of enzyme activity between males with missense vs. males with nonsense mutations was not feasible because only one male had a nonsense mutation (29 males with missense mutation vs. 1 male with nonsense mutations).

### Gb_3_ in plasma and urine and Lyso-Gb_3_ in plasma

Gb_3_ in plasma and in urine was available in 102 (36 males and 66 females) and 88 (34 males and 54 females) patients, respectively. Lyso-Gb_3_ in plasma was available in 84 patients (29 males and 55 females). Due to the use of different laboratories and assays during the reported period, the results were used only in relation to the overall assessment of each individual index-patient’s disease severity. Nevertheless, males had plasma Gb_3_ and lyso-Gb_3_ concentrations, respectively, higher than the normal upper limit (high vs normal plasma Gb_3_: 24 (67%) vs 12 (33%); high vs normal plasma lyso-Gb_3_: 25 (86%) vs 4 (14%)), while the opposite was true for the female Fabry patients (high vs normal plasma Gb_3_: 17 (26%) vs 49 (74%); high vs normal plasma lyso-Gb_3_: 22 (40%) vs 33 (60%)).

## Discussion

In this retrospective study we have presented data on genetic screening from the Danish Fabry Disease Centre at Department of Endocrinology and Metabolism at Copenhagen University Hospital, Rigshospitalet, which has served as national reference center for Fabry disease patients in the Kingdom of Denmark since 2001. The department is responsible for diagnosis and follow-up of adult Fabry disease patients as well as for genetic counseling and screening of their families while children with Fabry are followed in collaboration with the Centre for Inherited Metabolic Diseases at the same hospital. The Danish Fabry Disease Centre provides a comprehensive overview of Fabry disease patients’ medical history and health condition and constitutes a significant research tool for this rare disease with the help of the population-based National Patient Register, which contains detailed medical information on admissions to somatic hospital departments since 1977 [34]. More than 25 published papers have so far been based, totally or partially, on data from the Danish Fabry Disease Centre [26, 30, 35–50] including randomized clinical trials [51–55]. In this paper we aimed to report the 20 years of experience in diagnosing patients with Fabry disease in Denmark and describe the genetic counseling and cascade screening procedure conducted in their family members. Follow-up was done concerning age (calculated as mean) at baseline and end of the study period as well as status of dead or alive.

The vast majority of patients in the Danish cohort were identified after family cascade screening: 18 index-patients and 97 genetically identified family members with Fabry, or in mean 5.4 affected family members per index-case. This number is slightly higher than the one reported in a recent literature review on family genetic testing in Fabry disease, which revealed the identification of an average of 4.8 additional affected family members per index-patient (365 index-patients and 1744 affected family members) [56]. In addition, it indicates high efficacy of the cascade family screening for the identification of Fabry patients. Other valuable tools for the identification of undiagnosed Fabry patients are newborn screening or screening of at-risk populations (e.g., hypertrophic cardiomyopathy, hemodialysis patients, stroke). It has been suggested that the combination of the cascade family screening with the newborn/at-risk populations screening could identify several additional undiagnosed Fabry patients [56, 57]. However, screenings conducted in newborns or in at-risk populations carry a high risk of identifying numerous likely benign and benign *GLA* variants [56].

Most of the families in this study were of the classical Fabry genotype, but those referred from genetic variant screening programs for e.g. hypertrophic cardiomyopathy are mostly of later onset types and of milder phenotypes [56, 58]. The great majority of the variants identified in our cohort were unique (’private’). Most of the alleles were missense variants (15/24) and we found in addition 4 nonsense variants, 1 large deletion, 1 indel (blend of insertion and deletion), and three single nucleotide deletions/duplications affecting splice sites or reading frame. The distribution reflects the overall reported pathogenic variants (HGMD Professional 2021.3 database): 67.7% missense/nonsense [59], fabry-database.org: 71% missense, 17% nonsense variants [19]. None of the missense variants were novel whereas one nonsense and four of the remaining variants were novel. Four of the novel variants are loss-of-function variants (large deletion, deletion-frameshift, splice site deletion exon 6 skipping, duplication-frameshift) and the index-cases harbouring them were males with confirmed phenotypic Fabry disease metabolic defects. Therefore, these four variants have been interpretated as pathogenic according to the American College of Medical Genetics and Genomics and the Association for Molecular Pathology guidelines [60]. The fifth novel variant is a nonsense variant, and the index-patient was a female with normal enzyme activity, high concentrations of plasma lyso-Gb_3_, hypertrophic cardiomyopathy (endomyocardial biopsy revealed Fabry disease specific abnormalities) but without manifestations from the kidney. No male family member harbouring the variant has been identified. This variant can be interpretated as at least likely pathogenic (class 4) as a loss-of-function variant. It should be noted that in general, nonsense variants are associated with the classic phenotype [61].

In X-linked inheritance disorders, the number of heterozygotes is expected to be the double of homozygotes. In our cohort, we identified twice as many heterozygotes as homozygotes. This underlines the completeness of the family screening. Nevertheless, because Fabry disease is an X-linked recessive disorder, the heterozygotes present with a heterogenous phenotypic spectrum, from asymptomatic to as severely affected as their male relatives. A possible explanation for this wide clinical spectrum may be a skewed X chromosome inactivation, which results in preferential inactivation of the X chromosome carrying the normal allele [62].

Index-patients were referred to the Department from other clinics, mainly from cardiology where they had been diagnosed during genetic investigation for cardiomyopathy [41]; to a lesser extent, from neurologists due to atypical neuropathic pain or migraine, or from nephrologists due to incidental findings in a kidney biopsy. In some countries many of the Fabry patients with first manifestations from a stroke or neuropathic pain would be identified by screening [63, 64]. A few countries perform newborn screening [65], which has resulted in disclosing many variants of unknown significance in the *GLA* gene and only few patients with classical Fabry disease. None of these screenings have been performed in Denmark.

The heterogeneity and the shared clinical manifestations of Fabry disease with other diseases often result in delayed diagnosis. Cascade genetic screening in known families is a simple and effective method for early diagnosis of the disease.

Early diagnosis of treatable rare genetic diseases is important when effective therapies exist, that can delay the progression of the disease and improve outcomes. Previous studies have shown that Fabry disease patients with classical *GLA* variants that have initiated treatment in early stages had better outcomes compared with those having started treatment later, especially when irreversible organ damage was already present [66–70]. Moreover, early diagnosis provides the opportunity to offer follow-up in a multidisciplinary and genetic counseling team [56]. Systematic and proper cascade screening requires thorough genetic counseling of all family members. In young Fabry patients it is important to counsel early to be able to provide them with a good basis for reproductive choices, including information about the inheritance pattern, recurrence risks, the individually different phenotype/genotype relationships and the availability of preimplantation and prenatal genetic diagnostics.

Performing proper genetic and management counseling also requires a thorough knowledge of the phenotype/genotype relationship as well as treatment options and their possible outcomes in relation to the individual phenotype of the patient. In the beginning of the era of the first enzyme replacement therapy, it was still not recognized that females could be as affected of Fabry disease as males. It was also not recognized that a severely affected individual could have an asymptomatic child and vice versa–this was only gradually published [71, 72].

In our Fabry cohort, two confirmed index-cases refused further investigation (and follow-up) and in addition one of them refused to provide information on close family members. In general, index-cases, as well as the family members identified via family screening were overwhelmingly compliant to inform their relatives. There is an ongoing discussion about patients’ “right not to know” and “right to refuse treatment”. Arguments defending and criticizing the “right not to know” are based on the values of autonomy, liberty and duty [73]. One can argue that patients should take responsibility for decisions regarding the management of their health and lives enacting their “right not to know” and “right to refuse treatment”, however, it should not be neglected that this could negatively affect their family members that might carry the same pathogenic variant, and thus deprive their family members of their “right to know”.

The mean age of death in our cohort was 47.0 years for males and 64.8 years for females, compared with a life expectancy at birth (2000–2020) of 74.3–79.6 and 79.0–83.6 years, respectively, in the general population in Denmark [74]. The mean age of death revealed in our cohort was lower in males but higher in females than the one reported in the Fabry Fabrazyme^®^ Registry (53.7 years in males and 60.9 years in females) [75]. This could be due to a number of factors, small cohorts with low statistical power being the most likely. Data from the same registry revealed that the most common cause of death in Fabry disease patients was cardiovascular disease, followed by cerebrovascular events (i.e. stroke). Unfortunately, in our cohort the cause of death was not known in all deceased patients.

### Strengths and limitations

To the extent of our knowledge, we present one of the most complete and unbiased genetic cascade screenings in a nationwide Fabry cohort. All adult Danish patients with Fabry were followed in a single center, which ensures internal consistency across all examinations. More importantly all relevant family members were offered genetic testing once a Fabry positive diagnosis had been made and complete organ assessment was performed in all patients irrespective of symptoms. A strength of the study is that we have verified our unbiased genetic inclusion by having twice the number of females in the national cohort compared to males.

Due to suboptimal compliance issues some index-cases and family members lacked the initial assessments which is one of the limitations. Furthermore, given that Gb_3_ measurements were not available in 2001, these were initiated later and subsequently obtained from two laboratories, both of which changed methodology over time. Finally, a substantial data amount was collected prior to the lyso-Gb_3_ era why most patients did not have these measurements initially.

### Conclusions and perspectives

In this paper, we present descriptive outcomes of 20 years of experience in diagnosis, mortality and cascade screening of Fabry disease patients in the Danish Reference Centre for Fabry disease, resulting in as complete family screening as possible, taking into consideration consent from patients, by including twice the number of females as males due to X-linked inheritance. The completeness of the Fabry patient identification in the country has as such resulted in a cohort of balanced genotypes according to gender, indicating that the cohort was not biased by referral, and further resulted in earlier diagnosis of the disease by a lower age at diagnosis in family members compared to index-patients; such is rarely seen in other Fabry cohorts [66, 76]. Six previously unreported disease-causing variants in the *GLA* gene were discovered.

This nationwide screening and registration of Fabry disease families provides a unique possibility to establish a complete cohort of Fabry patients and to advance current knowledge of this rare lysosomal storage inherited disorders.

## Supporting information

S1 Data (SAV)

## References

[1] Brady RO, Gal AE, Bradley RM, Martensson E, Warshaw AL, Laster L. Enzymatic Defect in Fabry’s Disease. New England Journal of Medicine. 1967 May;276(21):1163–7. 6023233 10.1056/NEJM196705252762101

[2] Schiffmann R. Fabry disease. Pharmacology & Therapeutics. 2009 Apr;122(1):65–77. 19318041 10.1016/j.pharmthera.2009.01.003

[3] Fabry J. Ein Beitrag zur Kenntniss der Purpura haemorrhagica nodularis (Purpura papulosa haemorrhagica Hebrae). Archiv für Dermatologie und Syphilis. 1898 Dec;43(1):187–200.

[4] Anderson W. A case of “angeio-keratoma.” British Journal of Dermatology. 1898 Apr;10(4):113–7.

[5] Pompen AWM, Ruiter M, Wyers HJG. Angiokeratoma corporis diffusum (universale) Fabry, as a sign of an unknown internal disease; two autopsy reports. Acta Medica Scandinavica. 2009 Apr;128(3):234–55. doi: 10.1111/j.0954-6820.1947.tb06596.x 18897399

[6] Kint JA. Fabry’s Disease: Alpha-Galactosidase Deficiency. Science. 1970 Feb;167(3922):1268–9. doi: 10.1126/science.167.3922.1268 5411915

[7] Sweeley CC, Klionsky B. Fabry’s Disease: Classification as a Sphingolipidosis and Partial Characterization of a Novel Glycolipid. Journal of Biological Chemistry. 1963 Sep;238(9):PC3148–50. 14081947

[8] Krivit W, Desnick RJ, Bernlohr RW, Wold F, Najarian JS, Simmons RL. Enzyme Transplantation in Fabry’s Disease. New England Journal of Medicine. 1972 Dec;287(24):1248–9. doi: 10.1056/NEJM197212142872411 4563680

[9] Bishop DF, Calhoun DH, Bernstein HS, Hantzopoulos P, Quinn M, Desnick RJ. Human alpha-galactosidase A: nucleotide sequence of a cDNA clone encoding the mature enzyme. Proceedings of the National Academy of Sciences. 1986 Jul;83(13):4859–63. doi: 10.1073/pnas.83.13.4859 3014515 PMC323842

[10] Bishop DF, Kornreich R, Desnick RJ. Structural organization of the human alpha-galactosidase A gene: further evidence for the absence of a 3’ untranslated region. Proceedings of the National Academy of Sciences. 1988 Jun;85(11):3903–7. doi: 10.1073/pnas.85.11.3903 2836863 PMC280328

[11] Bernstein HS, Bishop DF, Astrin KH, Kornreich R, Eng CM, Sakuraba H, et al. Fabry disease: six gene rearrangements and an exonic point mutation in the alpha-galactosidase gene. Journal of Clinical Investigation. 1989 Apr;83(4):1390–9. doi: 10.1172/JCI114027 2539398 PMC303833

[12] Christensen Lou, H. O. Angiokeratoma Corporis Diffusum (Fabry’s Sygdom). Copenhagen, Polyteknisk Forlag. 1972.

[13] Galjaard H, Niermeiwr MF, Hahnemann N, Mohr J, Sørensen SA. An example of rapid prenatal diagnosis of Fabry’s disease using microtechniques. Clinical Genetics. 2008 Apr;5(4):368–77. 10.1111/j.1399-0004.1974.tb01708.x 4211797

[14] Madsen KM, Hasholt L, Sørensen SA, van Loo A, Vanholder R. The utility of single-strand conformation polymorphism (SSCP) analysis: Results obtained in families with Fabry’s disease. Scandinavian Journal of Clinical and Laboratory Investigation. 1996 Jan;56(2):177–82. doi: 10.3109/00365519609088605 8743111

[15] Feldt-Rasmussen U, Rasmussen AK, Mersebach H, Rosenberg KM, Hasholt L, Sorensen SA. Fabry disease: a new challenge in endocrinology and metabolism? European Journal of Endocrinology. 2002 Jun;741–2. doi: 10.1530/eje.0.1460741 12039692

[16] Roberts MC, Dotson WD, DeVore CS, Bednar EM, Bowen DJ, Ganiats TG, et al. Delivery Of Cascade Screening For Hereditary Conditions: A Scoping Review Of The Literature. Health Affairs. 2018 May;37(5):801–8. doi: 10.1377/hlthaff.2017.1630 29733730 PMC11022644

[17] National Cancer Institute. NCI Dictionaries. https://www.cancer.gov/publications/dictionaries/genetics-dictionary/def/cascade-screening. Accessed November 20, 2021.

[18] Dobyns W. The pattern of inheritance of X-linked traits is not dominant or recessive, just X-linked. Acta Paediatrica. 2006 Apr;95(0):11–5. doi: 10.1080/08035320600618759 16720459

[19] fabry-database. http://fabry-database.org Accessed November 16, 2021.

[20] International Fabry Disease Genotype- Phenotype Database http://dbfgp.org/dbFgb/fabry/ Accessed November 16, 2021.

[21] Danmarks Statistik. Befolkningstal. https://www.dst.dk/da/Statistik/emner/befolkning-og-valg/befolkning-og-befolkningsfremskrivning/folketal. Accessed November 16, 2021.

[22] Facts about Greenland—Naalakkersuisut. https://naalakkersuisut.gl/en/About-government-of-greenland/About-Greenland/Facts-about-Greenland. Accessed November 16, 2021.

[23] The official gateway to the Faroe Islands. Faroe Islands population https://www.faroeislands.fo/people-society/people-of-the-faroe-islands/population/ Accessed November 16, 2021.

[24] Danish Council of Ethics Genome testing—Ethical dilemmas in diagnosis, in research and direct-to-consumer https://www.etiskraad.dk/~/media/Etisk-Raad/en/Publications/Genome-testing-report-2012.pdf?la=da Accessed June 1, 2022.

[25] Madsen KM, Hasholt L, Sørensen SA, Fermér ML, Dahl N. Two novel mutations (L32P) and (G85N) among five different missense mutations in six Danish families with Fabry’s disease. Human Mutation. 1995;5(3):277–8. doi: 10.1002/humu.1380050316 7599642

[26] Feldt-Rasmussen U, Dobrovolny R, Nazarenko I, Ballegaard M, Hasholt L, Rasmussen ÅK, et al. Diagnostic dilemma: A young woman with Fabry disease symptoms, no family history, and a “sequencing cryptic” α-galactosidase a large deletion. Molecular Genetics and Metabolism. 2011 Nov;104(3):314–8. doi: 10.1016/j.ymgme.2011.05.008 21641253

[27] Shabbeer J, Yasuda M, Benson SD, Desnick RJ. Fabry disease: Identification of 50 novel α-galactosidase A mutations causing the classic phenotype and three-dimensional structural analysis of 29 missense mutations. Human Genomics. 2006;2(5):297. 16595074 10.1186/1479-7364-2-5-297 PMC3500179

[28] Dobrovolny R, Nazarenko I, Kim J, Doheny D, Desnick RJ. Detection of large gene rearrangements in X-linked genes by dosage analysis: identification of novel α-galactosidase A (GLA) deletions causing Fabry disease. Human Mutation. 2011 Mar;32(6):688–95. doi: 10.1002/humu.21474 21305660

[29] Dobrovolny R, Dvorakova L, Ledvinova J, Magage S, Bultas J, Lubanda JC, et al. Relationship between X-inactivation and clinical involvement in Fabry heterozygotes. Eleven novel mutations in the α-galactosidase A gene in the Czech and Slovak population. Journal of Molecular Medicine. 2005 Apr;83(8):647–54. 15806320 10.1007/s00109-005-0656-2

[30] Feldt-Rasmussen U, Hasholt L, Ballegaard M, Christiansen M, Law I, Lund A, et al. The D313Y variant in the GLA gene—no evidence of a pathogenic role in Fabry disease in 2 Danish families. Molecular Genetics and Metabolism. 2018 Feb;123(2):S44.

[31] Desnick RJ, Allen KY, Desnick SJ, Raman MK, Bernlohr RW, Krivit W. Fabry’s disease: enzymatic diagnosis of hemizygotes and heterozygotes. Alpha-galactosidase activities in plasma, serum, urine, and leukocytes. J Lab Clin Med. 1973 Feb;81(2):157–71. 4683418

[32] Apelland T, Gude E, Strøm EH, Gullestad L, Eiklid KL, Månsson JE, et al. Familial globotriaosylceramide-associated cardiomyopathy mimicking Fabry disease. Heart. 2014 Jul;100(22):1793–8. doi: 10.1136/heartjnl-2014-305616 25031264

[33] Roddy TP, Nelson BC, Sung CC, Araghi S, Wilkens D, Zhang XK, et al. Liquid chromatography–tandem mass spectrometry quantification of globotriaosylceramide in plasma for long-term monitoring of Fabry patients treated with enzyme replacement therapy. Clinical Chemistry. 2005 Jan;51(1):237–40. doi: 10.1373/clinchem.2004.038323 15514097

[34] The National Patient Register. https://econ.au.dk/the-national-centre-for-register-based-research/danish-registers/the-national-patient-register/browse. Accessed November 20, 2021.

[35] Effraimidis G, Feldt-Rasmussen U, Rasmussen ÅK, Lavoie P, Abaoui M, Boutin M, et al. Globotriaosylsphingosine (lyso-Gb3) and analogues in plasma and urine of patients with Fabry disease and correlations with long-term treatment and genotypes in a nationwide female Danish cohort. Journal of Medical Genetics. 2020 Sep;58(10):692–700. doi: 10.1136/jmedgenet-2020-107162 32963035

[36] Madsen CV, Granqvist H, Petersen JH, Rasmussen ÅK, Lund AM, Oturai P, et al. Age-related renal function decline in Fabry disease patients on enzyme replacement therapy: a longitudinal cohort study. Nephrology Dialysis Transplantation. 2018 Dec;34(9):1525–33. 10.1093/ndt/gfy357 30535327

[37] Prabakaran T, Birn H, Bibby BM, Regeniter A, Sorensen SS, Feldt-Rasmussen U, et al. Long-term enzyme replacement therapy is associated with reduced proteinuria and preserved proximal tubular function in women with Fabry disease. Nephrology Dialysis Transplantation. 2013 Nov;29(3):619–25. doi: 10.1093/ndt/gft452 24215016

[38] Prabakaran T, Nielsen R, Satchell SC, Mathieson PW, Feldt-Rasmussen U, Sørensen SS, et al. Mannose 6-phosphate receptor and sortilin mediated endocytosis of α-galactosidase A in kidney endothelial cells. Deli MA, editor. PLoS ONE. 2012 Jun;7(6):e39975. doi: 10.1371/journal.pone.0039975 22768187 PMC3386966

[39] Prabakaran T, Nielsen R, Larsen JV, Sørensen SS, Rasmussen UF, Saleem MA, et al. Receptor-mediated endocytosis of α-galactosidase A in human podocytes in Fabry disease. Dussaule JC, editor. PLoS ONE. 2011 Sep;6(9):e25065. doi: 10.1371/journal.pone.0025065 21949853 PMC3176300

[40] Madsen CV, Bundgaard H, Rasmussen ÅK, Sørensen SS, Petersen JH, Køber L, et al. Echocardiographic and clinical findings in patients with Fabry disease during long-term enzyme replacement therapy: a nationwide Danish cohort study. Scandinavian Cardiovascular Journal. 2017 May;51(4):207–16. doi: 10.1080/14017431.2017.1332383 28545342

[41] Havndrup O, Christiansen M, Stoevring B, Jensen M, Hoffman-Bang J, Andersen PS, et al. Fabry disease mimicking hypertrophic cardiomyopathy: genetic screening needed for establishing the diagnosis in women. European Journal of Heart Failure. 2010 Jun;12(6):535–40. doi: 10.1093/eurjhf/hfq073 20498269

[42] Korsholm K, Feldt-Rasmussen U, Granqvist H, Højgaard L, Bollinger B, Rasmussen AK, et al. Positron emission tomography and magnetic resonance imaging of the brain in Fabry disease: a nationwide, long-time, prospective follow-up. PLOS ONE. 2015 Dec;10(12):e0143940. doi: 10.1371/journal.pone.0143940 26629990 PMC4667906

[43] Yazdanfard PD, Madsen CV, Nielsen LH, Rasmussen ÅK, Petersen JH, Seth A, et al. Significant hearing loss in Fabry disease: Study of the Danish nationwide cohort prior to treatment. PLOS ONE. 2019 Dec;14(12):e0225071. doi: 10.1371/journal.pone.0225071 31809513 PMC6897399

[44] Yazdanfard PDW, Effraimidis G, Madsen CV, Nielsen LH, Rasmussen ÅK, Petersen JH, et al. Hearing loss in Fabry disease: A 16 year follow-up study of the Danish nationwide cohort. Molecular Genetics and Metabolism. 2021 Feb;132(2):S114. 10.1016/j.ymgmr.2022.100841 PMC8857513 35242579

[45] Fledelius HC, Sandfeld L, Rasmussen ÅK, Madsen CV, Feldt-Rasmussen U. Ophthalmic experience over 10 years in an observational nationwide Danish cohort of Fabry patients with access to enzyme replacement. Acta Ophthalmologica. 2014 Dec;93(3):258–64. doi: 10.1111/aos.12588 25487570

[46] Torvin Møller A, Winther Bach F, Feldt-Rasmussen U, Rasmussen Å, Hasholt L, Lan H, et al. Functional and structural nerve fiber findings in heterozygote patients with Fabry disease. Pain. 2009 Sep;145(1):237–45. doi: 10.1016/j.pain.2009.06.032 19665302

[47] Moller AT, Feldt-Rasmussen U, Rasmussen AK, Sommer C, Hasholt L, Bach FW, et al. Small-fibre neuropathy in female Fabry patients: reduced allodynia and skin blood flow after topical capsaicin. Journal of the Peripheral Nervous System. 2006 Jun;11(2):119–25. doi: 10.1111/j.1085-9489.2006.00076.x 16787509

[48] Møller AT, Bach FW, Feldt-Rasmussen U, Rasmussen ÅK, Hasholt L, Sommer C, et al. Autonomic skin responses in females with Fabry disease. Journal of the Peripheral Nervous System. 2009 Sep;14(3):159–64. doi: 10.1111/j.1529-8027.2009.00227.x 19909479

[49] Borgwardt L, Feldt-Rasmussen U, Rasmussen AK, Ballegaard M, Meldgaard Lund A. Fabry disease in children: agalsidase-beta enzyme replacement therapy. Clinical Genetics. 2012 Sep;83(5):432–8. doi: 10.1111/j.1399-0004.2012.01947.x 22880956

[50] Madsen CV, Christensen EI, Nielsen R, Mogensen H, Rasmussen ÅK, Feldt-Rasmussen U. Enzyme replacement therapy during pregnancy in Fabry patients: Review of published cases of live births and a new case of a severely affected female with Fabry Disease and pre-eclampsia complicating pregnancy. JIMD Rep. 2019;44:93–101. Available from: doi: 10.1007/8904_2018_129 30117110 PMC6323029

[51] Bichet DG, Torra R, Wallace E, Hughes D, Giugliani R, Skuban N, et al. Long-term follow-up of renal function in patients treated with migalastat for Fabry disease. Molecular Genetics and Metabolism Reports. 2021 Sep;28:100786. doi: 10.1016/j.ymgmr.2021.100786 34401344 PMC8353473

[52] Hughes DA, Nicholls K, Sunder-Plassmann G, Jovanovic A, Feldt-Rasmussen U, Schiffmann R, et al. Safety of switching to Migalastat from enzyme replacement therapy in Fabry disease: Experience from the Phase 3 ATTRACT study. American Journal of Medical Genetics Part A. 2019 Mar;179(6):1069–73. doi: 10.1002/ajmg.a.61105 30920142 PMC6593787

[53] Schiffmann R, Bichet DG, Jovanovic A, Hughes DA, Giugliani R, Feldt-Rasmussen U, et al. Migalastat improves diarrhea in patients with Fabry disease: clinical-biomarker correlations from the phase 3 FACETS trial. Orphanet Journal of Rare Diseases [Internet]. 2018 Apr;13(1). Available from: doi: 10.1186/s13023-018-0813-7 29703262 PMC5923014

[54] Feldt-Rasmussen U, Hughes D, Sunder-Plassmann G, Shankar S, Olivotto I, Ortiz D, et al. Oral pharmacological chaperone migalastat compared with enzyme replacement therapy in Fabry disease: 30-month results from the randomized phase 3 ATTRACT study. Molecular Genetics and Metabolism. 2019 Feb;126(2):S53.

[55] Germain DP, Hughes DA, Nicholls K, Bichet DG, Giugliani R, Wilcox WR, et al. Treatment of Fabry’s Disease with the pharmacologic Chaperone Migalastat. New England Journal of Medicine. 2016 Aug;375(6):545–55. doi: 10.1056/NEJMoa1510198 27509102

[56] Germain DP, Moiseev S, Suárez-Obando F, Al Ismaili F, Al Khawaja H, Altarescu G, et al. The benefits and challenges of family genetic testing in rare genetic diseases-lessons from Fabry disease. Mol Genet Genomic Med. 2021 May;9(5):e1666. doi: 10.1002/mgg3.1666 33835733 PMC8172211

[57] Doheny D, Srinivasan R, Pagant S, Chen B, Yasuda M, Desnick RJ. Fabry Disease: prevalence of affected males and heterozygotes with pathogenic GLA mutations identified by screening renal, cardiac and stroke clinics, 1995–2017. J Med Genet. 2018 Apr;55(4):261–8. doi: 10.1136/jmedgenet-2017-105080 29330335

[58] Maron MS, Xin W, Sims KB, Butler R, Haas TS, Rowin EJ, et al. Identification of Fabry Disease in a Tertiary Referral Cohort of Patients with Hypertrophic Cardiomyopathy. Am J Med. 2018 Feb;131(2):200.e1–200.e8. 10.1016/j.amjmed.2017.09.010 28943383

[59] The Human Gene Mutation Database www.hgmd.cf.ac.uk/ac/index.php Accessed November 20, 2021.

[60] Richards S, Aziz N, Bale S, Bick D, Das S, Gastier-Foster J, et al. Standards and guidelines for the interpretation of sequence variants: a joint consensus recommendation of the American College of Medical Genetics and Genomics and the Association for Molecular Pathology. Genetics in Medicine. 2015 May;17(5):405–24. doi: 10.1038/gim.2015.30 25741868 PMC4544753

[61] Ortiz A, Germain DP, Desnick RJ, Politei J, Mauer M, Burlina A, et al. Fabry disease revisited: Management and treatment recommendations for adult patients. Molecular Genetics and Metabolism. 2018 Apr;123(4):416–27. doi: 10.1016/j.ymgme.2018.02.014 29530533

[62] Viggiano E, Politano L. X Chromosome Inactivation in carriers of Fabry disease: Review and Meta-Analysis. Int J Mol Sci. 2021 Jul 17;22(14):7663. doi: 10.3390/ijms22147663 34299283 PMC8304911

[63] Ortiz JF, Parwani J, Millhouse PW, Eissa-Garcés A, Hassen G, Cuenca VD, et al. Prevalence of Fabry disease in patients with cryptogenic strokes: a systematic review. Cureus. 2021 Nov;13(11):e19358. doi: 10.7759/cureus.19358 34925972 PMC8654093

[64] Tomek A, Petra R, Paulasová Schwabová J, Olšerová A, Škorňa M, Nevšímalová M, et al. Nationwide screening for Fabry disease in unselected stroke patients. PLoS One. 2021;16(12):e0260601. doi: 10.1371/journal.pone.0260601 34905550 PMC8670679

[65] van der Tol L, Smid BE, Poorthuis BJHM, Biegstraaten M, Deprez RHL, Linthorst GE, et al. A systematic review on screening for Fabry disease: prevalence of individuals with genetic variants of unknown significance. J Med Genet. 2014 Jan;51(1):1–9. doi: 10.1136/jmedgenet-2013-101857 23922385

[66] Germain DP, Charrow J, Desnick RJ, Guffon N, Kempf J, Lachmann RH, et al. Ten-year outcome of enzyme replacement therapy with agalsidase beta in patients with Fabry disease. J Med Genet. 2015 May;52(5):353–8. doi: 10.1136/jmedgenet-2014-102797 25795794 PMC4413801

[67] Germain DP, Arad M, Burlina A, Elliott PM, Falissard B, Feldt-Rasmussen U, et al. The effect of enzyme replacement therapy on clinical outcomes in female patients with Fabry disease–A systematic literature review by a European panel of experts. Molecular Genetics and Metabolism. 2019 Mar;126(3):224–35. doi: 10.1016/j.ymgme.2018.09.007 30413388

[68] Ortiz A, Abiose A, Bichet DG, Cabrera G, Charrow J, Germain DP, et al. Time to treatment benefit for adult patients with Fabry disease receiving agalsidase β: data from the Fabry Registry. J Med Genet. 2016 Jul;53(7):495–502. doi: 10.1136/jmedgenet-2015-103486 26993266 PMC4941144

[69] Wanner C, Arad M, Baron R, Burlina A, Elliott PM, Feldt-Rasmussen U, et al. European expert consensus statement on therapeutic goals in Fabry disease. Molecular Genetics and Metabolism. 2018 Jul;124(3):189–203. doi: 10.1016/j.ymgme.2018.06.004 30017653

[70] Warnock DG, Ortiz A, Mauer M, Linthorst GE, Oliveira JP, Serra AL, et al. Renal outcomes of agalsidase beta treatment for Fabry disease: role of proteinuria and timing of treatment initiation. Nephrology Dialysis Transplantation. 2012 Mar 1;27(3):1042–9. doi: 10.1093/ndt/gfr420 21804088 PMC3289896

[71] Eng CM, Germain DP, Banikazemi M, Warnock DG, Wanner C, Hopkin RJ, et al. Fabry disease: guidelines for the evaluation and management of multi-organ system involvement. Genet Med. 2006 Sep;8(9):539–48. doi: 10.1097/01.gim.0000237866.70357.c6 16980809

[72] Wilcox WR, Oliveira JP, Hopkin RJ, Ortiz A, Banikazemi M, Feldt-Rasmussen U, et al. Females with Fabry disease frequently have major organ involvement: lessons from the Fabry Registry. Mol Genet Metab. 2008 Feb;93(2):112–28. doi: 10.1016/j.ymgme.2007.09.013 18037317

[73] Davies B, Savulescu J. The Right Not to Know: some Steps towards a Compromise. Ethic Theory Moral Prac. 2021 Mar;24(1):137–50. 10.1007/s10677-020-10133-9 PMC7611423 34335078

[74] OECD, Policies EO on HS and. Denmark: Country Health Profile 2021 [Internet]. 2021. https://www.oecd-ilibrary.org/content/publication/2dce8636-en

[75] Waldek S, Patel MR, Banikazemi M, Lemay R, Lee P. Life expectancy and cause of death in males and females with Fabry disease: Findings from the Fabry Registry. Genetics in Medicine. 2009 Sep;11(11):790–6. doi: 10.1097/GIM.0b013e3181bb05bb 19745746

[76] Eng CM, Fletcher J, Wilcox WR, Waldek S, Scott CR, Sillence DO, et al. Fabry disease: baseline medical characteristics of a cohort of 1765 males and females in the Fabry Registry. J Inherit Metab Dis. 2007 Apr;30(2):184–92. doi: 10.1007/s10545-007-0521-2 17347915

