# Correction: Systematic cascade screening in the Danish Fabry Disease Centre: 20 years of a national single-centre experience

**DOI:** 10.1371/journal.pone.0287268

**Published:** 2023-06-08

**Authors:** Grigoris Effraimidis, Åse Krogh Rasmussen, Morten Dunoe, Lis F. Hasholt, Flemming Wibrand, Soren S Sorensen, Allan M. Lund, Lars Kober, Henning Bundgaard, Puriya D. W. Yazdanfard, Peter Oturai, Vibeke A. Larsen, Victor Hugo Fraga de Abreu, Lotte Hahn Enevoldsen, Tatiana Kristensen, Kirsten Svenstrup, Margrethe Bastholm Bille, Farah Arif, Mette Mogensen, Mads Klokker, Vibeke Backer, Caroline Kistorp, Ulla Feldt-Rasmussen

The thirteenth author’s initials are indexed incorrectly in PubMed. The correct initials are: Fraga de Abreu VH.
